# Corticosteroid Treatment for Leptospirosis: A Systematic Review and Meta-Analysis

**DOI:** 10.3390/jcm13154310

**Published:** 2024-07-24

**Authors:** Pavlo Petakh, Valentyn Oksenych, Oleksandr Kamyshnyi

**Affiliations:** 1 Department of Biochemistry and Pharmacology, Uzhhorod National University, 88000 Uzhhorod, Ukraine; pavlo.petakh@uzhnu.edu.ua; 2 Department of Microbiology, Virology and Immunology, I. Horbachevsky Ternopil National Medical University, 46001 Ternopil, Ukraine; 3 Broegelmann Research Laboratory, Department of Clinical Science, University of Bergen, 5020 Bergen, Norway

**Keywords:** leptospirosis, corticosteroids, mortality, mechanical ventilation, acute respiratory distress syndrome

## Abstract

**Background:** Leptospirosis, a zoonotic disease prevalent in tropical regions, often leads to severe complications such as Weil’s disease and acute respiratory distress syndrome (ARDS). This pioneering meta-analysis investigated the role of corticosteroids in treating severe leptospirosis, addressing a critical gap in the current clinical knowledge. **Methods:** We systematically reviewed studies from PubMed and Scopus, focusing on randomized controlled trials and observational cohort studies involving adult patients diagnosed with leptospirosis. Five studies comprising 279 participants met the inclusion criteria. **Results:** Although some studies suggest potential benefits, particularly for pulmonary complications, the evidence remains inconclusive due to the limited number of studies and their methodological limitations. Notably, while four of the five reviewed studies indicated a possible positive role of corticosteroids, the single randomized controlled trial showed no significant benefit, highlighting the need for more robust research. **Conclusions:** While the current evidence provides a basis for potential benefits, it is not sufficient to make definitive clinical recommendations. Further research is essential to clarify the role of corticosteroids in the treatment of severe leptospirosis, with the aim of improving patient outcomes and guiding clinical practices effectively.

## 1. Introduction

Leptospirosis, a disease primarily found in tropical regions, is a zoonotic illness transmitted through direct or indirect contact with the urine of animals, particularly rats [1]. Leptospirosis is estimated to cause approximately 1.03 million cases and 58,900 deaths annually [2]. The majority of these cases and fatalities are found in adult males aged 20–49 years [2]. The highest rates of illness and death occur in the Global Burden of Disease (GBD) regions of south and southeast Asia, Oceania, the Caribbean, Andean Latin America, central and tropical Latin America, and eastern sub-Saharan Africa.

Pathogenic Leptospira, which cause the disease, are excreted in the urine of infected animals, leading to humans inadvertently becoming hosts and potentially facing life-threatening outcomes [3]. Rats, however, do not suffer fatal infections and act as natural reservoirs. Most human infections with Leptospira are mild or asymptomatic. When symptoms do occur, they usually begin suddenly and include fever, chills, muscle aches, headache, and other flu-like symptoms [4,5].

Diagnosing leptospirosis poses significant challenges, particularly in clinical settings with limited resources. Its clinical symptoms are non-specific and resemble those of other tropical infectious diseases. Diagnostic methods include polymerase chain reaction (PCR) techniques and serological tests, with the microscopic agglutination test (MAT) being the most commonly used and considered to be the gold standard [6]. However, these diagnostic tests are often constrained by their availability and the expenses associated with maintaining laboratory standards. Specifically, MAT requires the continuous upkeep of bacterial cultures and demonstrates a lower sensitivity during the acute phase of the disease. While PCR tests offer a greater sensitivity, they are not commonly available or utilized in regions where the disease is highly endemic. An effective laboratory diagnosis of leptospirosis necessitates a combination of diagnostic techniques and appropriate sample collection, tailored to the disease stage and available resources [7].

Leptospirosis is treated with antibiotics, including doxycycline, azithromycin, cephalosporins, or penicillin [8]. However, the effectiveness of antibiotic therapy, especially in severe cases, is still uncertain. Treating spirochetal infections like leptospirosis with antibiotics can lead to the Jarisch–Herxheimer reaction, which involves symptoms such as shaking chills, fever, the worsening of skin rashes, and, in rare instances, multi-organ failure [9].

In recent years, there has been a surge in research aimed at identifying novel synthetic and natural compounds with sporicidal activity against Leptospira species. For example, Ishak et al. (2019) reported the sporicidal activity of extracts from *Canarium odontophyllum* leaves, known locally as dabai in Sarawak and kembayau in Sabah and Brunei, highlighting the therapeutic potential of natural remedies [10]. Arulmozhi et al. investigated the ethanolic extract of *Andrographis paniculata* leaves, commonly known as creat or green chiretta, and found it to possess sporicidal activity against various *Leptospira* species, suggesting its potential as an alternative treatment [11]. Additionally, probiotic bacteria and dietary supplements have the potential to prevent or reverse antibiotic-associated gut microbiota dysbiosis in patients with leptospirosis [12]. Not only do antibiotics affect the gut microbiota, but leptospira infection itself also alters the gut microbiota. Research by Xie et al. (2022) revealed significant changes in microbial composition, particularly an increased Firmicutes/Bacteroidetes ratio, following infection [13].

The typical pattern of leptospirosis is described as “biphasic” [14]. The initial phase involves an acute period of fever and bacteremia lasting from 2 to 9 days, followed by a phase where fever subsides and patients may appear to improve. The second phase, known as the “immune” phase, is characterized by a return of fever and the onset of complications. Approximately 5% to 15% of patients may progress to Weil’s disease, which often includes pulmonary involvement, affecting between 20% and 70% of cases [3]. Pulmonary complications can range from mild cough to severe symptoms such as hemoptysis and acute respiratory distress syndrome (ARDS), with the latter having a high mortality rate of around 50%. Additionally, severe pulmonary hemorrhage syndrome (SPHS) due to leptospirosis has reported mortality rates ranging from 50% to 70% [1,15,16,17].

Host responses in leptospirosis play a crucial role in its pathogenesis [18]. Contact with the pathogen triggers the release of cytokines, including interleukin 6 (IL-6), interleukin 1 beta (IL-1β), and tumor necrosis factor-alpha (TNF-α) [19,20,21]. The extensive release of these cytokines is known as a cytokine storm [22]. Multiple studies and systematic reviews have found that the cytokine storm occurring during the second (immune) phase is a major factor contributing to the severity of leptospirosis [21,23]. Indika Senavirathna et al., in their systematic review, reported that the levels of IL-1β, IL-2, IL-4, IL-6, IL-8, IL-10, and TNF-α were significantly higher in severe cases of leptospirosis compared to mild cases [24]. When comparing the antibody responses between individuals with severe and mild leptospirosis, it was found that over 74% of those in the severe group showed a notable rise in immunoglobulin (Ig)G levels, whereas this increase was less pronounced in the mild group [25].

A similar cytokine storm is often observed in patients with COVID-19, leading to severe disease progression and acute respiratory distress syndrome (ARDS), with a higher mortality rate [26,27]. To treat severe COVID-19 accompanied by a cytokine storm, immunomodulatory agents like corticosteroids, such as dexamethasone, are widely used. Corticosteroids have anti-inflammatory and immunosuppressive effects that reduce the production of pro-inflammatory cytokines like IL-6 [28]. Previous studies have shown improved prognoses and survival rates in patients with moderate to severe COVID-19 using corticosteroid therapy, including dexamethasone, methylprednisolone, and hydrocortisone [29,30]. The RECOVERY trial, a randomized controlled study involving 6425 hospitalized COVID-19 patients, found that those treated with dexamethasone alongside standard therapy had a lower mortality rate (22.9%) compared to those who received only standard treatment (25.7%) [31]. Additionally, a study by Ohoud Aljuhani et al. found that dexamethasone resulted in a lower multiple organ dysfunction syndrome (MODS) score on the third day of ICU admission compared to methylprednisolone, although there was no statistically significant difference in COVID-19 mortality [32].

The role of corticosteroids in treating severe leptospirosis, especially in addressing pulmonary complications like ARDS, has been explored in a limited number of studies. One argument posits that multi-organ failure in leptospirosis may result from an overactive immune system rather than the direct effects of the pathogen [21]. Therefore, the use of therapeutic doses of steroids is considered to counteract immune activation, potentially reducing mortality and morbidity in severe leptospirosis cases [33]. Corticosteroids are also considered for potentially reducing the frequency or intensity of the Jarisch–Herxheimer reaction [34].

Overall, more recent studies have demonstrated the favorable benefits of corticosteroids in treating COVID-19 and similar severe acute respiratory diseases. However, the efficacy of corticosteroids remains uncertain due to the limited number of studies. Therefore, the objective of this systematic review and meta-analysis is to assess the effectiveness of corticosteroids in treating leptospirosis. Given the worldwide prevalence and mortality potential of leptospirosis, this review is crucial for helping clinicians to understand the evidence related to the benefits and harms of corticosteroid treatment in leptospirosis patients.

## 2. Materials and Methods

### 2.1. Data Sources and Search Strategy

**Figure 1:**
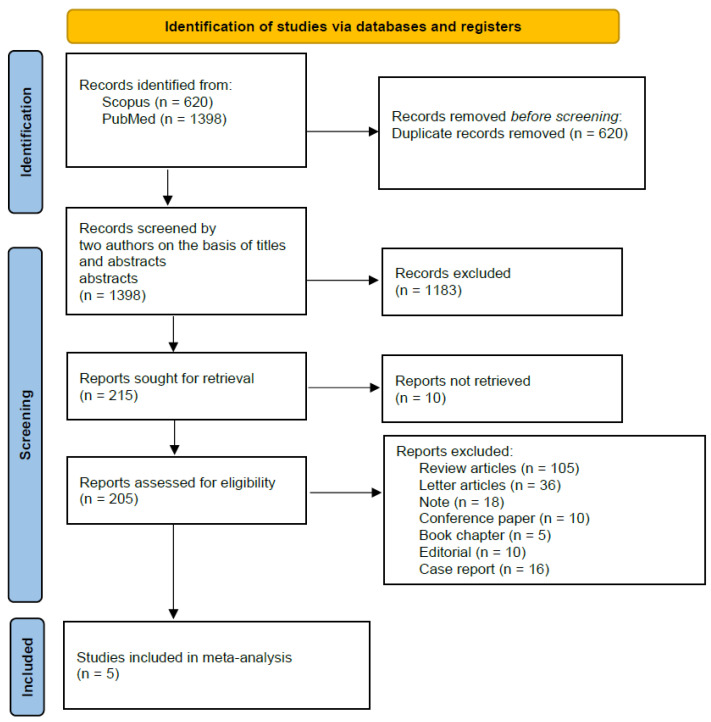
PRISMA flow diagram. In total, we identified 2018 scientific records through databases. In the end, only 5 met the inclusion criteria for the meta-analysis.

This systematic review, conducted in accordance with the PRISMA guidelines, involved a comprehensive search for published studies in PubMed and Scopus from 1 January 1948 to 1 October 2023 (Figure 1) [35]. The search strategy, detailed in Table 1, combined terms related to corticosteroids and leptospirosis, ensuring comprehensive coverage of relevant publications. The review protocol was registered and can be accessed via the specified identifier on the CRD website https://www.crd.york.ac.uk, identifier CRD42024508820 (accessed on 23 June 2024).

### 2.2. Eligibility

Randomized controlled trials (RCTs) and observational cohort studies investigating the impact of corticosteroids on leptospirosis were considered eligible if they satisfied the following inclusion criteria: the inclusion of adult patients (age ≥ 18 years), the confirmation of leptospirosis diagnosis through PCR, MAT, or ELISA, the provision of outcome measures related to corticosteroid treatment, and no restrictions on the type, dose, and duration of corticosteroids. Studies involving pregnant women or children, reviews, case reports, and articles not available in English were excluded.

### 2.3. Definition of Primary and Secondary Outcomes

The primary outcome was all-cause hospital mortality (the quantity of survivors and non-survivors among those who were and were not administered corticosteroids). The secondary outcome was mechanical ventilation (i.e., as defined by the study: the need for invasive mechanical ventilation, the duration of mechanical ventilation, ventilator-free days, or other oxygen therapy).

### 2.4. Study Selection and Quality Analysis

Two independent reviewers evaluated the titles and abstracts of all the identified records to determine their eligibility for inclusion in the meta-analysis. Any disagreements between the reviewers were resolved through discussion and consensus. The same reviewers then retrieved and assessed the full-text articles of the potentially eligible studies to make their final inclusion decisions. Data from the selected studies were entered into Microsoft Excel (Microsoft Office Professional Plus 2019) version 1809, considering the following information for each study: year of publication, study design, subjects treated with corticosteroids (and those not treated), and clinical outcomes regarding the effectiveness of corticosteroids. The quality and reliability of each study were independently assessed using the Risk Of Bias In Non-randomised Studies—of Interventions (ROBINS-I) tool (Version 1 August 2016) [36].

The evaluation considered biases arising from confounding, the selection of participants for the study, the classification of interventions, deviations from the intended interventions, missing data, the measurement of the outcome, and the selection of the reported result. The response options for each risk of bias assessment included: a low risk of bias, a moderate risk of bias, a serious risk of bias, a critical risk of bias, and no information.

### 2.5. Heterogeneity

We evaluated heterogeneity using Q-tests and measured the proportion of total variability attributable to heterogeneity with the I^2^ statistic. An I^2^ value of less than 50% was classified as a low heterogeneity, values between 50% and 74% as a medium heterogeneity, and values of 75% or higher as a high heterogeneity. Significant heterogeneity indicates that the study characteristics were substantially different.

### 2.6. Assessment of Evidence Quality

The evidence certainty was evaluated by two reviewers using the Grading of Recommendations Assessment, Development, and Evaluation (GRADE) framework. The quality of evidence was downgraded based on five domains: risk of bias, inconsistency, indirectness, imprecision, and publication bias. Overall, the evidence certainty was categorized as very low, low, moderate, or high [37].

### 2.7. Statistical Analysis

The data were entered into Microsoft Excel, and a meta-analysis was conducted using the software Comprehensive Meta-Analysis V3, employing a random-effects model [38]. Efficacy summary measures, expressed as odds ratios (OR) along with their corresponding 95% confidence intervals (CI), were assessed.

## 3. Results

### 3.1. Study Selection

Five prospective studies comprising a total of 279 participants were included in the meta-analysis. All selected studies were conducted in the Asian region. The characteristics of the patients, along with details regarding the type of corticosteroids administered and their respective regimens, are delineated in Table 2. Notably, in 80% of the studies (four out of five), methylprednisolone was the most frequently prescribed corticosteroid. According to the criteria for the laboratory diagnosis of leptospirosis from the five included studies, two studies—Ittiachen et al. [39] and Shenoy et al. [40]—were identified as having a higher risk of bias. Ittiachen et al. [39] did not state how they documented the diagnosis of leptospirosis in patients, while Shenoy et al. [40] used a rapid dipstick test for lepto IgM, confirmed by an IgM ELISA test. These methods are considered to have a higher risk of bias compared to the other studies that used MAT or PCR tests.

**Figure 2:**
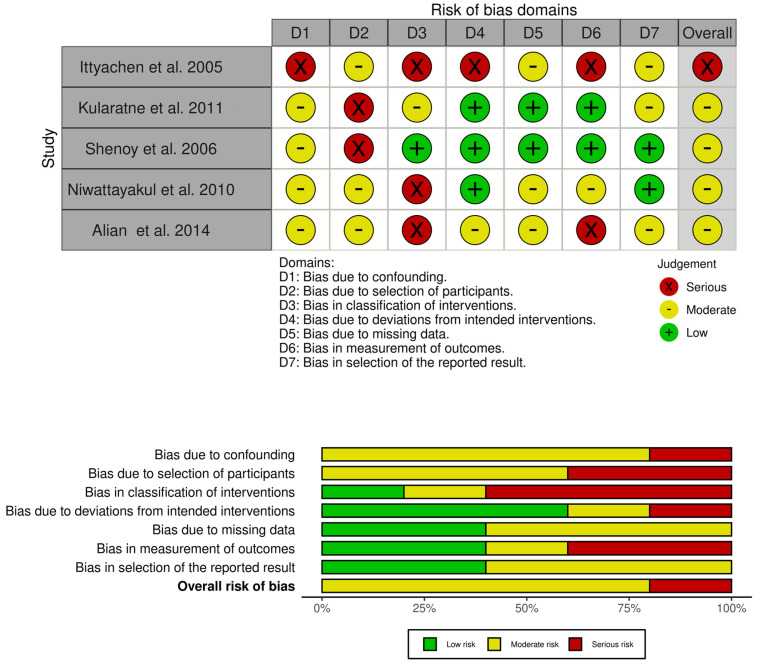
Risk of bias. Generated using ROBINS−I. Most articles had a moderate risk of bias, except for one article, which had a serious risk of bias [39,40,41,42,43].

All of the included studies were prospective, which increases the risk of bias and results in a lower level of evidence, as confirmed by the GRADE classification (Table 1, Figure 2).

### 3.2. Effect of Steroids on Primary and Secondary Outcomes

#### 3.2.1. Mortality

**Figure 3:**
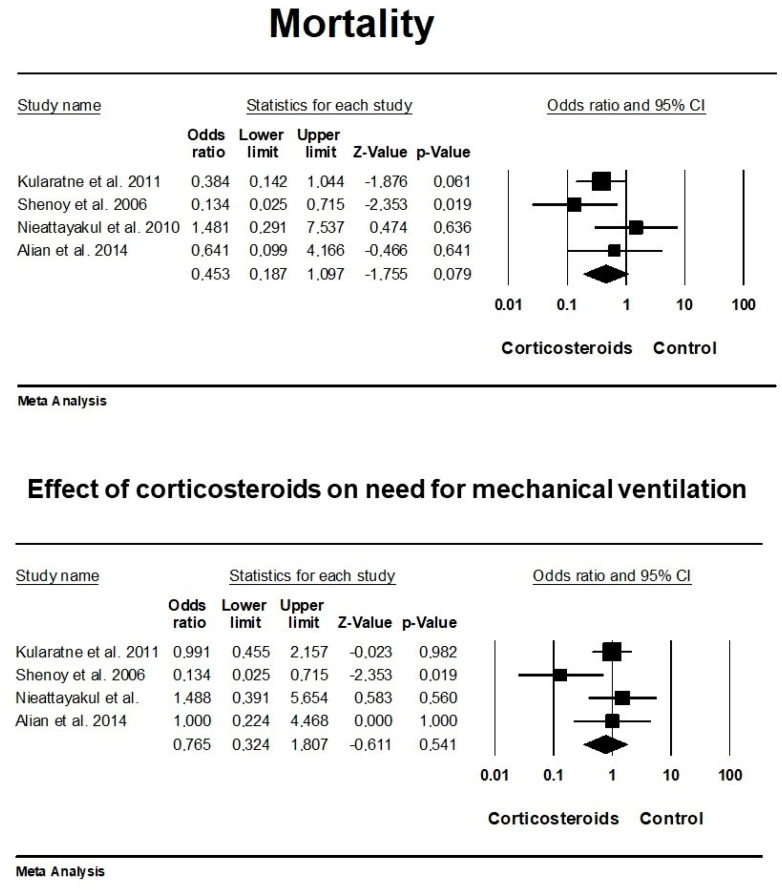
Effect of corticosteroids on mortality and need for mechanical ventilation [40,41,42,43].

In all the included studies, comprehensive data on mortality rates were available. However, the study conducted by Ittyachen et al. (2005) [39] was excluded from our meta-analysis due to the absence of a control group, precluding a comparative assessment. The reported mortality rate in this particular study was 12.5%. Across the remaining studies, the mortality rate was consistently higher in the control groups without corticosteroid intervention, except for the study by Nieattayakul et al. (2010) [42]. In this study, the mortality rate in the control group was 13%, compared to a slightly higher rate of 18% observed in the dexamethasone-treated group. The overall risk estimate (OR) was 0.453 (95% CI: 0.18–1.09), indicating a potential beneficial effect of corticosteroid use in patients with leptospirosis on mortality. However, it is noteworthy that the observed difference between the effects of corticosteroids in the treated group and the control regarding mortality did not reach statistical significance (*p* = 0.079). The included studies demonstrated a moderate level of heterogeneity, as indicated by an overall I^2^ value of 30.29% (*p* = 0.230). The between-study variance (tau^2^) was calculated to be 0.251 (Figure 3).

#### 3.2.2. Requirement of Mechanical Ventilation

The meta-analysis incorporated data from four studies concerning the requirement for mechanical ventilation due to respiratory insufficiency. The study conducted by Ittyachen et al. (2005) [39] was excluded from this analysis due to data unavailability. Among the included studies, two exhibited an equal number of patients: Kularatne et al. (2011) [41] and Alian et al. (2014) [43]. In one study, a slightly higher proportion of patients who were prescribed corticosteroids required mechanical ventilation (77% vs. 69%), while in another study, the reverse trend was observed, with a lower proportion in the corticosteroid group requiring ventilation (17% vs. 61%). The overall risk estimate (OR) was 0.765 (95% CI: 0.32–1.80), suggesting a potential beneficial effect of corticosteroid use in patients with leptospirosis on the incidence of mechanical ventilation for respiratory insufficiency. Nevertheless, it is noteworthy that the observed difference between the effects of corticosteroids in the treated group and the control regarding the incidence of mechanical ventilation for respiratory insufficiency did not achieve statistical significance (*p* = 0.541). The included studies exhibited a moderate level of heterogeneity, as evidenced by an overall I^2^ value of 45.93% (*p* = 0.136). The between-study variance (tau^2^) was calculated to be 0.349 (Figure 3).

## 4. Discussion

This is the first meta-analysis aimed at investigating the role of corticosteroids in the treatment of leptospirosis, particularly its severe forms such as Weil’s disease and ARDS. To date, only a systematic review by Rodrigo et al. and the 2022 Cochrane Hepato-Biliary Group meta-analysis protocol have been conducted [44,45].

Based on the currently available evidence, a definitive recommendation regarding the use of corticosteroids for the treatment of severe leptospirosis remains elusive. The limited number of studies and their methodological shortcomings contribute to the challenge of drawing conclusive findings. Among the identified five studies, four suggested a potential beneficial role of steroids, particularly in patients with lung involvement. However, it is crucial to note that these four studies are characterized as prospective case series, with one having a single-arm design and the remaining three comparing the corticosteroid group with a historical cohort. The lone randomized controlled trial, while inconclusive due to statistical underpowering, did not demonstrate a significant benefit of steroids in severe leptospirosis. Moreover, the use of corticosteroids was associated with an increased risk of nosocomial infections across all studies.

Moderate bias was observed in all reviewed studies, further complicating the interpretation of their results. Additionally, the heterogeneity in the treatment regimens adds another layer of complexity. In three studies, methylprednisolone was administered at the initiation of treatment, but at varying doses. The study employing dexamethasone at the initiation failed to show a treatment benefit and, notably, reported an elevated incidence of nosocomial infections [42]. The dose of dexamethasone in this particular study was comparatively high relative to the equivalent doses of MP used in other investigations, introducing further variability in the assessment of the treatment outcomes.

The randomized controlled trial employing the pulse methylprednisolone protocol instills considerable optimism regarding the efficacy of prednisolone compared to a placebo in the treatment of pulmonary involvement associated with severe leptospirosis [ISRCTN74625030] (Azevedo et al., 2011) [46]. This trial, registered in 2011, boasts a substantial sample size and a robust level of evidence. Despite our efforts to acquire data by reaching out to the authors, regrettably, we did not receive a response.

Numerous reports highlighting the benefits of steroid administration in the management of severe Weil’s disease have emanated primarily from case studies [44,47,48,49,50,51]. The latest study, conducted in 2022, undertook a comparative analysis between COVID-19 and leptospirosis [14].

Jayakrishnan et al. reported recovery in a patient with severe pulmonary leptospirosis treated with intravenous methylprednisolone (i.v. MP) [52]. Minor et al. documented recovery in a patient with severe leptospirosis and acute kidney injury treated with i.v. MP, without antibiotics [48]. Montero-Tinnirello et al. described a fatal case of severe pulmonary leptospirosis despite treatment with i.v. MP [53]. Thunga et al. noted recovery in a patient with severe pulmonary leptospirosis treated with i.v. MP [54]. Maroun et al. also reported recovery in a patient with severe pulmonary leptospirosis treated with i.v. MP [55]. Meaudre et al. described a case of severe leptospirosis with acute kidney injury and rhabdomyolysis, where the patient recovered after treatment with i.v. MP and intravenous immunoglobulins [56]. Turhan et al. documented a fatal case of severe pulmonary leptospirosis despite i.v. MP treatment [57]. Lawrence et al. reported recovery in a patient with severe leptospirosis treated with high-dose intravenous steroids, though the specific steroid was unidentified [58]. Courtin et al. also described recovery in a patient with severe leptospirosis treated with unidentified high-dose intravenous steroids [59]. Kingscote et al. noted recovery in a patient with severe leptospirosis, including acute kidney injury and hepatitis, treated with intravenous hydrocortisone [60]. Despite being distinct diseases, both exhibited comparable life-saving responses to steroid treatment, with the shared pathogenic factor identified as the cytokine storm [14].

## 5. Conclusions

This meta-analysis is the first to explore the efficacy of corticosteroids in treating severe leptospirosis, including Weil’s disease and ARDS. Despite some studies suggesting potential benefits, particularly for pulmonary complications, the evidence remains inconclusive due to the limited number of studies and their methodological limitations. Notably, while four of the five reviewed studies indicated a possible positive role for corticosteroids, the only randomized controlled trial did not show significant benefits, highlighting the need for more robust research.

The findings indicate that corticosteroid use in leptospirosis is associated with a risk of nosocomial infections, which further complicates the assessment of their overall benefit. The variability in treatment regimens, particularly in the doses and types of corticosteroids used, adds another layer of complexity in interpreting the results.

Given the parallels in the immune response mechanisms between leptospirosis and other severe acute respiratory diseases like COVID-19, where corticosteroids have shown favorable outcomes, there is a rationale for continued investigation into their use for leptospirosis. However, the moderate bias and heterogeneity in the existing studies underscore the urgent need for well-designed, randomized controlled trials to definitively determine the efficacy and safety of corticosteroids in this context.

In conclusion, while current evidence provides a foundation for potential benefits, it is not sufficient to make definitive clinical recommendations. Further research is essential to clarify the role of corticosteroids in the management of severe leptospirosis, aiming to improve patient outcomes and guide clinical practices effectively.

## 6. Future Research Directions

The current body of evidence underscores the imperative for more robust research to definitively establish the efficacy and safety of corticosteroids in severe leptospirosis. Future research should focus on well-designed RCTs with larger sample sizes, stratifying patients based on disease severity and pulmonary complications, and including appropriate control groups to compare corticosteroid efficacy with standard care or other immunomodulatory treatments. The implementation of blinding techniques will reduce bias and enhance result reliability. Future directions include:

Investigating corticosteroid mechanisms in modulating the immune response in leptospirosis, focusing on cytokine profiles, inflammatory markers, and other immunological parameters. Exploring interactions between Leptospira and the host immune system to understand disease progression modulation by corticosteroids.

Comparing the efficacy and safety of different corticosteroids (e.g., dexamethasone, methylprednisolone, and prednisolone) and exploring combination therapies with antibiotics, antivirals, or other immunomodulatory agents.

Conducting long-term follow-up studies to evaluate the sustained impact of corticosteroid therapy on survival, quality of life, and chronic complication incidence among leptospirosis survivors. Monitoring and reporting on long-term adverse effects, particularly secondary infections and immunosuppressive-related complications. Investigating corticosteroid efficacy across diverse geographical regions with varying Leptospira strains and patient demographics, focusing on vulnerable populations such as immunocompromised individuals, elderly patients, and those with comorbidities.

## Figures and Tables

**Table 1:** Search strategy.

Database	Search Terms
Scopus	TITLE-ABS-KEY (corticosteroid* OR corticoid* OR glucocortico* OR hydrocortison* OR hydroxycorticosteroid* OR prednisolon* OR prednison* OR betamethason* OR dexamethason* OR beclomethason* OR methylprednisolon* OR “adrenal cortex hormon*” OR steroid* OR hydroxypregnenolon* OR tetrahydrocortisol* OR cortodoxon* OR cortison* OR fludrocortison* OR corticosteron* OR paramethason* OR cortisol* OR triamcinolon*) AND TITLE-ABS-KEY (leptospir* OR ((weil* OR “Swineherd*”) AND disease*) OR “Stuttgart disease*” OR “hemorrhagic jaundice” OR “spirochetal jaundice” OR ((“cane cutter” OR canicola OR icterohemorrhagic OR mud OR “rice field” OR swamp) AND fever))
PubMed	(corticosteroid* OR corticoid* OR glucocortico* OR hydrocortison* OR hydroxycorticosteroid* OR prednisolon* OR prednison* OR betamethason* OR dexamethason* OR beclomethason* OR methylprednisolon* OR “adrenal cortex hormon*” OR steroid* OR hydroxypregnenolon* OR tetrahydrocortisol* OR cortodoxon* OR cortison* OR fludrocortison* OR corticosteron* OR paramethason* OR cortisol* OR triamcinolon*) AND (leptospir* OR ((weil* OR “Swineherd*”) AND disease*) OR “Stuttgart disease*” OR “hemorrhagic jaundice” OR “spirochetal jaundice” OR ((“cane cutter” OR canicola OR icterohemorrhagic OR mud OR “rice field” OR swamp) AND fever))

**Table 2:** Characteristics of included studies.

Author, Year	Study Design	Country	Certainty of Evidence (Grade)	No of Participants	Type of Corticosteroids and Dosage Regimen
Ittyachen et al. 2005 [39]	Prospective study	India	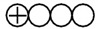	8	Methylprednisolone (40 mg every 8 h)
Kularatne et al. 2011 [41]	Prospective study	Sri Lanka	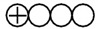	140	Methylprednisolone 500 mg given for 3 days followed by 8 mg orally for 5 days
Shenoy et al. 2006 [40]	Prospective study	India	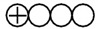	30	Methylprednisolone 1 g/day for three days followed by 1 mg/kg/day of oral prednisolone for 7 days
Niwattayakul et al. 2010 [42]	Prospective open randomized controlled trial	Thailand	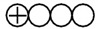	45	Dexamethasone once daily for 3 days followed by 1 mg/kg/day oral prednisolone for 4 days
Alian et al. 2014 [43]	Prospective case–control studies	Iran	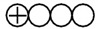	56	Prednisolone 1 mg/kg/day for maximum one week

## Data Availability

The raw data supporting the conclusions of this article will be made available by the authors on request.
